# Conservative Management of Spontaneous Isolated Dissection of the Superior Mesenteric Artery

**DOI:** 10.1155/2017/9623039

**Published:** 2017-07-16

**Authors:** Jae Hyun Kwon, Yoon Hee Han, Jun Kyu Lee

**Affiliations:** ^1^Department of Radiology, Dongguk University Ilsan Hospital, Dongguk University Graduate School of Medicine, 27 Dongguk-ro, Siksa-dong, Ilsandong-gu, Goyang-si, Gyeonggi-do 10326, Republic of Korea; ^2^Department of Internal Medicine, Dongguk University Ilsan Hospital, Dongguk University Graduate School of Medicine, 27 Dongguk-ro, Siksa-dong, Ilsandong-gu, Goyang-si, Gyeonggi-do 10326, Republic of Korea

## Abstract

**Purpose:**

We report the clinical outcomes of patients with spontaneous isolated dissection of the superior mesenteric artery (SIDSMA) who were treated conservatively.

**Materials and Methods:**

A retrospective review was performed in 14 patients from 2006 to 2016 with SIDSMA. Their clinical features and computed tomographic angiography (CTA) characteristics, treatment methods, and clinical outcomes were analyzed. The mean age was 53.6 (range, 41–73) years, and the mean follow-up duration was 20.6 (range, 1–54) months. Conservative management was the primary treatment if no bowel ischemia or arterial rupture was noted.

**Results:**

The mean initial abdominal visual analog pain score was 7 (range, 5–9) in seven patients. The mean total duration of abdominal pain was 10.2 days (range, 2–42 days) in 10 patients. The mean percentage stenosis of the dissected SMA at the initial presentation was 78.8% in 14 patients. Complete obstruction of the SMA at the initial presentation was evident in 4 of the 14 patients (28.6%). Conservative management was successful in all 14 patients. None of the 14 patients developed bowel ischemia or an infarction. Abdominal pain did not recur in any patient during follow-up (mean, 20.6 months; range, 1–54 months).

**Conclusion:**

Conservative management was successful for all SIDSMA patients, even those with severe compression of the true lumen or complete obstruction of the dissected SMA.

## 1. Introduction

Spontaneous isolated dissection of the superior mesenteric artery (SIDSMA) has recently been discovered more frequently because of the development of multidetector computed tomography (MDCT) [1, 2]. However, the natural clinical and morphological course of SIDSMA has yet not been established nor has a treatment method [3–11]. As a result, there is no established consensus treatment for the management of SIDSMA [4, 7, 9, 11–13]. In this study, we performed a retrospective analysis of the clinical course of patients with SIDSMA, managed conservatively.

## 2. Materials and Methods

This study was approved by the institutional review board (IRB) and the ethics committee of our institution. Informed consent was waived by the IRB because of the retrospective nature of the study.

Patients presenting with SIDSMA between July 2006 and February 2016 were enrolled in this retrospective study, resulting in a study population of 14 eligible patients with SIDSMA. We reviewed the electrical medical records (EMRs) and computed tomographic angiography (CTA) images of the 14 patients. We investigated demographic features (age and sex), clinical features (the severity and duration of abdominal pain, the mode of symptom onset, location, related symptoms, recurrence of abdominal pain, coexisting medical conditions, relation to dietary, and smoking history), lesion characteristics (type of dissection, dissection length, aneurismal change, stenotic degree of SMA, and morphological changes on follow-up CTA images), treatment method, and clinical outcomes at the end of follow-up (mean, 20.6 months; range, 2–54 months). The severity of abdominal pain at initial presentation and during follow-up was graded from 0 to 10 using a visual analog scale (VAS) in all patients. The time taken for relief from the initial severe abdominal pain was that required for the initial VAS score to fall to 1–3 during the admission and follow-up periods. The total duration of abdominal pain was defined as the period required for the initial abdominal pain to resolve completely.

The protocol used for CTA of the abdominal aorta was as follows: CTAs were performed using 64- and 128-channel MDCT (SOMATOM Sensation 64, SOMATOM Definition AS+; Siemens, Forchheim, Germany) scanners. Images were acquired from the diaphragm to the level of the ischial tuberosity using the following parameters: detector collimation 0.6; pitch 0.6; and gantry rotation time 0.5 s. Nonenhanced arterial and venous phase images were acquired. Arterial phase images were collected via automated bolus triggering; 80–120 mL nonionic contrast was injected at a rate of 4 mL/s. A circular region of interest (ROI) was placed in each upper abdominal aorta at the level of the celiac axis. CT acquisition was initiated when the ROI enhancement exceeded 100 HU after injection of contrast medium. Venous phase images were acquired 40 s after completion of the arterial phases. The raw data were reconstructed into axial images of both the nonenhanced and venous phases. Arterial phase images were reconstructed into axial, sagittal, and coronal images 2 mm in thickness. All data were then systematically analyzed using the maximum intensity projection (MIP), multiplanar reformatting (MPR), and three-dimensional volume rendering (3D-VR) of the various views, employing a dedicated workstation (Syngo CT Workplace; Siemens, Erlangen, Germany).

The diagnosis was made when one of the following signs was seen in the SMA on CT: (a) intimal flap and contrast enhancement within the false lumen (double-barreled dissection) or (b) crescent-shaped area along the wall of the SMA with higher attenuation than blood, showing no contrast enhancement after contrast material injection (dissection with thrombosed false lumen) [14]. Patients with concomitant aortic dissection, a history of blunt abdominal trauma, SMA catheterization, and a history of upper abdominal surgery were excluded from the study.

The SIDSMA was categorized into three types according to the presence of false lumen flow and true lumen patency at the dissected segment, as described by Yun et al. (Table 1) [15]. Aneurismal dilation was defined as a greater than 50% increase in the diameter of the SMA. Percentage stenosis of the true lumen in patients with thrombosis of the false lumen was determined by CTA based on the diameter of the region of the SMA with maximal stenosis between the origin of the SMA dissection and the most distal portion of the dissected segment of SMA. Percentage stenosis was calculated by dividing the smallest diameter of the true lumen of the SMA at the maximal stenosis point by the largest diameter of the same part of the SMA and multiplying the result by 100. Occlusion of the SMA was defined as an occlusion of any portion of the main trunk involved in the dissection. Two radiologists (JHK and YHH) with 10 and 12 years of clinical experience, respectively, interpreted the CT images. Differences in opinion were resolved by a discussion until a consensus was reached.

Conservative therapy was performed as the primary treatment if no bowel ischemia or arterial rupture was noted in the CTA images of the patients with symptomatic SIDSMA. Conservative treatments were observational therapy consisting of fasting, parenteral nutritional support, control of blood pressure, and medical therapy, including pain relief medications and anticoagulant or antithrombotic therapy. Anticoagulation therapy consisted of low-molecular-weight heparin (enoxaparin, 1 mg/kg twice daily) while the patient was hospitalized. After discharge, therapy consisted of an antiplatelet agent (e.g., 100 mg aspirin daily) without additional administration of an anticoagulant for 3–6 months. Endovascular intervention or surgical treatment was reserved for patients showing aggravation or no relief of abdominal pain, peritoneal irritation signs, or bowel infarction on CTA. The patients were followed in an outpatient department and underwent CTA for the evaluation of morphologic changes in dissected SMA. Follow-up CTA scans were performed every 3 months for 1 year and every year thereafter.

## 3. Results

The patients' demographic and clinical features are presented in Table 2. The mean VAS symptom severity score was 7 (range, 5–9) in 7 of 14 patients at the initial admission. In seven patients, symptom severity scores could not be evaluated because their SIDSMA had been diagnosed in other institutions prior to admission to our hospital (*n* = 2); an incidental diagnosis of SIDSMA in a patient with an intracranial hemorrhage (*n* = 1); and the unavailability of EMR data allowing evaluation of the VAS symptom severity score (*n* = 4). The durations required for improvement of the initial abdominal pain, and that required before the VAS score fell to 1–3, were available for 10 of the 14 patients. The mean duration was 2.6 (range, 1–14) days in the 10 patients. The times required for improvement of initial abdominal pain and that for the VAS score to fall to 1–3 were 1 day in seven patients, 2 days in one patient, 3 days in one patient, and 14 days in one patient. These times were not available for four patients in whom SIDSMA was incidentally found (*n* = 1); SIDSMA was diagnosed in another hospital (*n* = 1), and symptom severity was not evaluated in the EMR using the VAS (*n* = 2). The times taken for the initial severe abdominal pain to improve and resolve completely were available for 10 patients. The mean time was 10.2 (range, 2–42) days in the 10 patients. The times were not available for four patients in whom SIDSMA was diagnosed in another hospital (*n* = 1), for one in whom SIDSMA was incidentally diagnosed, and for two whose VAS scores had not been recorded in the EMR.

None of the 14 patients had dissection-related bowel ischemia or infarction nor was there any incident of recurrent abdominal pain during the follow-up period (mean, 20.6 months; range, 1–54 months).

Conservative treatment was successful as a primary treatment in 13 of 14 (92.9%) patients. Endovascular treatment was attempted in one patient who showed persistent severe abdominal pain despite conservative treatment. In this patient, percutaneous endovascular thrombectomy using a 6-Fr. vascular catheter (ENVOY Guiding Catheter; Codman, Randolph, MA, USA) was not successful because the guidewire could not cannulate into the true lumen and the amount of thrombus was too large to be removed by the catheter. Although the patient reported persistent abdominal pain, because there was no sign of peritoneal irritation and the SMA angiography revealed intact perfusion in the distal portion of the SMA and bowel loops via the communicating arcades, conservative management was applied again instead of surgical treatment. The symptoms of the patient were gradually relieved over 10 days with good recovery. Surgical treatment was not performed in any patient.

The CTA characteristics of the dissected SMAs are shown in Table 3. At the initial presentation, SIDSMA lesions were categorized into the following types in terms of the CTA findings, as described by Yun et al. [15]: type IIb, 10 cases (71.4%) and type III, four cases (28.6%). Complete obstruction of the false and true lumina of the SIDSMA was evident in 4 of 14 patients (28.6%) on the CTAs taken at the initial presentation. The mean percentage of stenosis of the dissected SMA was 78.8% in the 14 initial CTAs of the 14 patients. The mean length of the dissected SMA was 9.2 cm (range, 4–17 cm). No aneurismal change was evident in the CTAs taken at the initial presentation or during follow-up.

Follow-up CTAs were performed seven times in six patients within 2 weeks after the initial CTA. Complete obstruction of the dissected SMA was evident in two of the six patients (33.3%). These two patients also evidenced complete obstruction of the dissected SMA on their initial CTAs. The mean percentage stenosis of the dissected SMA was 87.3% on seven follow-up CTAs performed on six patients within 2 weeks. Follow-up CTAs were performed seven times on five patients 1–3 months after their initial CTAs. Complete obstruction of the dissected SMA was evident in two of the five (40%) patients. One of these patients exhibited a new complete obstruction of the dissected SMA attributable to aggravation of stenosis apparent on the initial CTA. The other patient also exhibited complete obstruction of the dissected SMA on the initial CTA. The mean percentage stenosis of the dissected SMA was 75.7% on seven follow-up CTAs performed on five patients 1–3 months after their initial CTA. Follow-up CTAs were performed three times on two patients 4–6 months after their initial CTAs. Complete obstruction of the dissected SMA was observed in both patients (100%). One of these patients exhibited a new complete obstruction of the dissected SMA attributable to aggravation of stenosis apparent on the initial CTA. The other patient also exhibited complete obstruction of the dissected SMA both initially and on the 1- and 3-month follow-up CTAs. Follow-up CTA was performed once on one patient 7–12 months after the initial CTA. This patient exhibited stenosis on the initial CTA and complete obstruction of the dissected SMA on the 4- to 6-month follow-up CTA.

**Figure 1:**
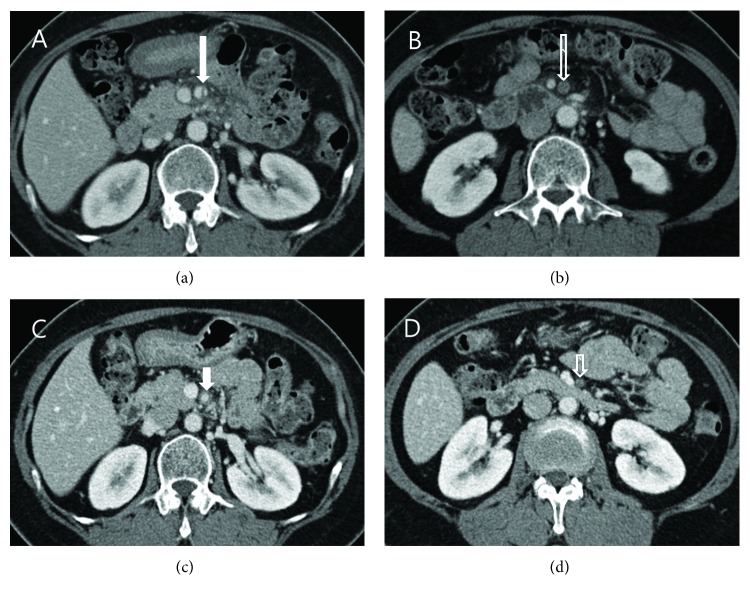
Computed tomographic angiography (CTA) findings in a 55-year-old woman with spontaneous isolated dissection of the superior mesenteric artery (SMA) at the initial presentation and at the 9-month follow-up exam. (a, b) Axial images show the dissection flap (long arrow) in the main trunk of the SMA and complete obstruction of the SMA, due to thrombosis (long empty arrow), at the initial presentation. (c, d) Axial CTA images at the 9-month follow-up exam show the SMA with a patent lumen at the proximal portion (short arrow) and a completely obstructed lumen at the midportion (short empty arrow).

CTAs evidencing complete obstruction or stenosis of the dissected SMA at initial presentation or during follow-up were not accompanied by any sign of bowel ischemia or infarction, such as bowel wall thickening, bowel dilation, or lack of bowel wall enhancement (Figure 1) [16].

## 4. Discussion

Potential etiologies of SIDSMA include atherosclerosis, cystic medial necrosis or degeneration of the arterial wall, trauma, mycosis, fibromuscular dysplasia, and inflammation [1, 12, 17, 18]. The potential risk factors include hypertension, smoking, hyperlipidemia, coronary heart disease, atherosclerosis, and diabetes mellitus [7, 19]. The exact pathophysiology of SIDSMA is not known; however, its preferential localization 2 to 6 cm after SMA origin should be favored by the abnormal mechanical stress on the anterior wall of the artery, between the fixed and mobile portions of the SMA at the pancreatic edge [12, 20, 21].

Recently, many studies have proposed treatment strategies for SIDSMA [3–7, 9, 11, 18, 22–24]. There are several treatment modalities for SIDSMA: conservative treatment, endovascular procedure, surgical repair, and bowel resection [3, 4, 9, 11, 13, 22, 23, 25–27]. Conservative management with or without anticoagulation for symptomatic SIDSMA has been reported to have a high clinical success rate, 90–95%, without recurrence of symptoms [15, 22, 23, 25]. It is not difficult to decide on either conservative management for asymptomatic patients or emergency operation for patients with bowel infarction or arterial rupture [2–4, 6, 7, 9, 11, 13–15, 22, 23, 25]. The indications for surgical treatment are arterial rupture, aneurismal dilation (>2 cm in diameter), bowel infarction, a positive peritoneal irritation sign, and progressive or persistent abdominal pain despite conservative treatment [2, 4, 9, 15]. However, there are controversies in the treatment strategy for patients with persistent abdominal pain despite conservative management and complete obstruction due to thrombosis or stenosis of the SMA true lumen [12, 15, 25, 26, 28, 29]. Causes of failure in conservative treatment include persistent abdominal pain, the development of aneurismal enlargement, a ruptured SMA aneurysm, and evidence of bowel ischemia or infarction [4, 15, 25].

Leung et al. [30] firstly reported endovascular stenting for the treatment of SIDSMA patients. Endovascular stenting is a useful treatment for persistent abdominal pain not relieved by conservative management and for severe stenosis or occlusion of the true lumen or aneurismal dilation (≥2.0 cm) [5, 9, 23, 30]. Several studies have suggested that the morphology (type, length, and severity of stenosis or obstruction) of the dissected SMA is important when endovascular stenting is contemplated in patients with SIDSMA [11, 18, 23, 30]. The primary or secondary endovascular stenting showed immediate pain relief and a shorter fasting time than conservative treatment without procedure-related complications [9, 10, 23]. These patients stayed asymptomatic, and follow-up CT scan revealed patent true lumen and stents, gradual resolution of the false lumen, reduced aneurysm size, and improved remodeling on a mid- to long-term follow-up periods [9, 23]. However, endovascular therapy is associated with certain risks and sometimes fails. Endovascular stenting of the superior mesenteric artery is associated with a higher risk of stent thrombosis than is stenting of other arteries. The long-term stability of stents in patients with SIDSMA has not yet been established [3, 27]. Long-term use of antithrombotic agents is essential, but no universally accepted protocol has yet been established [3, 7, 10, 23, 27].

Regarding the abdominal pain of the SIDSMA patient, the arterial dissection and mesenteric hematoma can be the cause of abdominal pain rather than ischemia, and it is difficult to diagnose intestinal ischemia based on symptoms alone [2, 8, 12]. An inflammatory response around the arterial dissection stimulating the visceral nerve plexus is known as a cause of abdominal pain [27, 31]. Longer dissection of the SMA could cause more perivascular inflammation and pain on the basis of the result that a positive correlation between pain severity and dissection length [15]. However, abdominal pain may be related to bowel ischemia or rupture of the dissected SMA, which needs endovascular or surgical treatment [4, 14, 19, 23, 32]. Persistent abdominal pain is an important factor to consider when selecting the treatment modality [6, 13, 18]. In our study, 10 patients who complained of severe abdominal pain at the initial presentation showed marked improvements in symptom severity (VAS score, 1–3) within a few days of conservative treatment. However, abdominal pain may persist for more than 1-2 weeks in patients who receive conservative management, as seen in our study and in other reports, although bowel ischemia was not apparent [12, 27]. In our opinion, persistent abdominal pain in patients receiving conservative treatment is not an important factor for deciding the treatment modality when no signs of bowel ischemic or infarction are present. In symptomatic patients, if there is no surgical indication, such as arterial rupture or bowel infarction, conservative management may be appropriate and can be continued even if these patients show persistent abdominal pain despite conservative management.

Many studies have reported a high clinical success rate of 90–95% without recurrence by conservative management with or without anticoagulation in symptomatic patients who show partial or complete thrombosis of a false lumen resulting in a steno-occlusive lesion in the SMA true lumen [12, 15, 18, 22, 23, 25, 27]. The causes of failure in conservative treatment were persistent abdominal pain, development aneurysmal enlargement, ruptured SMA aneurysm, and evidence of bowel ischemia or infarction [4, 15, 25]. Those patients in whom medical treatment failed were treated by endovascular or surgical treatment [4, 15, 25]. Dong et al. [13] showed that endovascular stent placement was attempted in nine patients with abdominal pain which did not subside during the first 3- to 5-day conservative treatment. The endovascular stent placement was successful in four patients and not successful in five patients. These five patients were successfully treated conservatively rather than surgical treatment, and their symptoms were gradually alleviated within the following week. Although presumption, if conservative treatment had been continued event if symptoms did not improve within 5 days, endovascular procedure might not have been necessary. In these studies, the severity of the luminal stenosis was not an important factor in the management strategy [12, 25, 27]. Severe stenosis or obstruction of the true lumen of the dissected SMA is not necessarily related to bowel ischemia or infarction [15, 22]. It was postulated that younger age, fewer risk factors, and comorbidities associated with atherosclerosis, and no evidence of systemic atherosclerosis compared to patients with other occlusive diseases of the SMA may be the cause of the discrepancy between the symptoms and stenosis or occlusion of the true lumen in SIDSMA [27]. Our patients with severe stenosis, or even obstruction of the dissected SMA, were managed successfully by conservative treatment. In this study, we found that steno-occlusive lesions of the true lumen of the SMA were not related to bowel ischemia or infarction. Prescription of anticoagulation therapy for SIDSMA patients can prevent false-lumen thrombosis at the dissected SMA. However, there is no evidence that anticoagulation therapy is of any value in SIDSMA patients [6, 29]. Conservative treatment with anticoagulation for SIDSMA patients was first reported by Ambo et al. in 1994 [33]. Since then, conservative treatment with anticoagulation for SIDSMA patients was reported in other literature with successful results [6, 14, 27, 29, 34]. For the rationale of using anticoagulation in treating SIDSMA is to prevent further hematoma formation of the stenotic segment and distal embolization even though the efficacy of conservative treatment with anticoagulation is similar to that without anticoagulation [7, 22]. The anticoagulation protocols (those of antithrombotic medication) used to treat SIDSMA patients vary among reports; a standard protocol is required [7, 15, 27].

The failure rate of conservative treatment, however, has been reported to be up to 16% because of the presence of aneurismal enlargement and bowel infarction [4, 23, 25]. Therefore, patients showing aggravation or persistent abdominal pain should be reevaluated for the presence of bowel infarction or aneurismal rupture [4, 25]. Diagnostic laparoscopy or laparotomy was suggested for patients with suspected bowel ischemia [18, 35]. In our opinion, a noninvasive diagnostic imaging method, such as MDCT, can be used to confirm the presence of bowel ischemia or arterial rupture, as in our study [36, 37]. MDCT shows high sensitivity and specificity for the diagnosis of intestinal ischemia [2, 36, 37]. If there is no bowel ischemia or rupture or enlargement of the aneurysm on MDCT, and no peritoneal irritation sign suggesting bowel ischemia, conservative management may be continued [3, 18, 27].

Our study had some limitations. First, it was retrospective in nature. Second, we included a small number of patients and there was no comparison among treatment modalities. Third, the follow-up duration was relatively short with respect to determining the development of aneurismal dilation or a chronic change in SIDSMA.

In conclusion, conservative management was successful in symptomatic patients with SIDSMA, even in those with persistent abdominal pain despite conservative management. In patients with steno-occlusive lesions in the true lumen of the dissected SMA, conservative management can be continued if there is no bowel ischemia, rupture, or enlargement of the aneurysm and no peritoneal irritation sign.

## Figures and Tables

**Table 1:** Categorization of angiographic findings of spontaneous isolated dissection of the superior mesenteric artery (SIDSMA).

Type	Angiographic findings
Type I	Patent true and false lumen revealing entry and reentry sites
Type II	Patent true lumen but no reentry flow from the false lumen
Type IIa	Visible false lumen but not visible reentry site (blind pouch of false lumen)
Type IIb	Not visible false luminal flow (thrombosed false lumen) which usually causes true lumen narrowing
Type III	SMA dissection with occlusion of SMA

**Table 2:** Patient demographics and clinical features with spontaneous isolated dissection of the superior mesenteric artery (*n* = 14).

Features	*n* (%)
Mean age (range), years	53.6 (41–73)
Follow-up (mean ± SD, range), months (*n* = 13)	20.6 ± 18.1 (1–54)
Male (*n*, %)	10 (71.4)
Pain	
Severity of initial pain (VAS, mean (range)) (*n* = 7)	7 (5–9)
Duration of initial severe pain (days, mean (range)) (*n* = 10)	2.6 (1–14)
Total duration of pain (days, mean (range)) (*n* = 10)	10.1 (2–42)
Onset mode	
Acute	11 (78.7)
Insidious	1 (7.1)
Incidental	1 (7.1)
N/A	1 (7.1)
Location	
Epigastric	8
Periumbilical	9
Not available	2
Other symptoms	
Nausea	3
Vomiting	3
Diarrhoea	3
Cold sweating	1
Radiating pain to back	2
Postprandial pain	2
Medical comorbidities and risk factors	
Diabetes mellitus	1
Hypertension	2
Hepatitis	1
Cerebrovascular disease	1
Hypothyroidism	1
Smoking (current and ex-smoker)	6

SD: standard deviation; NRS: numerical rating scale.

**Table 3:** CTA characteristics of spontaneous isolated dissection of the superior mesenteric artery.

		Initial CTA			F/U CTA within 2 weeks		F/U CTA at 1–3 months		F/U CTA at 4–6 months		F/U CTA at 7–12 months	
Number	Sex/age	Type	Stenosis (%)	TL/SMA dia. (mm)^∗^	Stenosis (%)	TL/SMA dia. (mm)^∗^	Stenosis (%)	TL/SMA dia. (mm)^∗^	Stenosis (%)	TL/SMA dia. (mm)^∗^	Stenosis (%)	TL/SMA dia. (mm)^∗^
1	M/55	IIb	67.1	2.7/8.2	—	—	0.0	4.5/4.5	—	—	—	—
2	M/49	IIb	76.4	2.1/8.9	—	—	—	—	—	—	—	—
3	M/54	III	100	0.0/8.8	—	—	—	—	—	—	—	—
4	M/41	IIb	68.2	2.7/8.5	—	—	—	—	100/100^†^	0.0/5.2, 0.0/5.2	100	0.0/6.0
5	F/57	IIb	79.5	1.7/8.3	—	—	69.5/100^†^	2.5/8.2, 0/6.6	—	—	—	—
6	F/73	IIb	71.3	2.7/9.4	—	—	—	—	—	—		—
7	M/50	IIb	57.5	3.1/7.3	75.0	1.8/7.2	75.6	3.0/12.3	—	—	—	—
8	F/55	III	100	0.0/10.5	—	—	100/100^†^	0.0/9.2, 0.0/4.6	100	0/5.6	—	—
9	M/60	III	100	0.0/9.7	100/100^†^	0/9.3, 0/10.9	—	—	—	—	—	—
10	M/56	IIb	74.2	2.3/8.9	80.6	2.1/10.8	—	—	—	—	—	—
11	M/47	IIb	75.4	3.4/13.8	76.1	3.4/14.2	85.1	2.0/13.4	—	—	—	—
12	F/55	IIb	76.3	2.8/11.8	79.5	2.3/11.2	—	—	—	—	—	—
13	M/54	III	100	0.0/8.7	100	0.0/8.6	—	—	—	—	—	—
14	M/45	IIb	57.3	5.0/11.7	—	—	—	—	—	—	—	—

CTA: computed tomographic angiography; F/U: follow-up; TL: true lumen; SMA: superior mesenteric artery; ^∗^diameter of the true lumen and superior mesenteric artery having most severely narrowed point; ^†^computed tomographic angiographs taken twice during follow-up periods.
